# Sex and disease severity-based analysis of steroid hormones in ME/CFS

**DOI:** 10.1007/s40618-024-02334-1

**Published:** 2024-05-10

**Authors:** Cornelia Pipper, Linda Bliem, Luis E. León, Daniela Mennickent, Claudia Bodner, Enrique Guzmán‑Gutiérrez, Michael Stingl, Eva Untersmayr, Bernhard Wagner, Romina Bertinat, Nuno Sepúlveda, Francisco Westermeier

**Affiliations:** 1 Institute of Biomedical Science, Department of Health Studies, FH Joanneum University of Applied Sciences, Graz, Austria; 2 Instituto de Ciencias Biomédicas, Facultad de Ciencias de La Salud, Universidad Autónoma de Chile, Santiago, Chile; 3 Departamento de Ciencias Básicas y Morfología, Facultad de Medicina, Universidad Católica de la Santísima Concepción, Concepción, Chile; 4 Machine Learning Applied in Biomedicine (MLAB), Concepción, Chile; 5 Departamento de Análisis Instrumental, Facultad de Farmacia, Universidad de Concepción, Concepción, Chile; 6 Departamento de Bioquímica Clínica e Inmunología, Facultad de Farmacia, Universidad de Concepción, Concepción, Chile; 7 Facharztzentrum Votivpark, Vienna, Austria; 8 Institute of Pathophysiology and Allergy Research, Center for Pathophysiology, Infectiology and Immunology, Medical University of Vienna, 1090 Vienna, Austria; 9 Centro de Microscopía Avanzada, CMA-BIO BIO, Facultad de Ciencias Biológicas, Universidad de Concepción, Concepción, Chile; 10 Faculty of Mathematics and Information Science, Warsaw University of Technology, Warsaw, Poland; 11 CEAUL – Centro de Estatística e Aplicações, Faculdade de Ciências, Universidade de Lisboa, Lisbon, Portugal; 12 Centro de Biología y Química Aplicada (CIBQA), Universidad Bernardo O’Higgins, Santiago, Chile

**Keywords:** Myalgic encephalomyelitis/chronic fatigue syndrome, Steroid hormones, Sex-related differences

## Abstract

**Purpose:**

Myalgic encephalomyelitis/chronic fatigue syndrome (ME/CFS) is a debilitating disease characterized by persistent fatigue and decreased daily activity following physical and/or cognitive exertion. While ME/CFS affects both sexes, there is a higher prevalence in women. However, studies evaluating this sex-related bias are limited.

**Methods:**

Circulating steroid hormones, including mineralocorticoids (aldosterone), glucocorticoids (cortisol, corticosterone, 11-deoxycortisol, cortisone), androgens (androstenedione, testosterone), and progestins (progesterone, 17α-hydroxyprogesterone), were measured in plasma samples using ultra-high performance liquid chromatography–tandem mass spectrometry (UHPLC–MS/MS). Samples were obtained from mild/moderate (ME/CFSmm; females, n=20; males, n=8), severely affected patients (ME/CFSsa; females, n=24; males, n=6), and healthy controls (HC, females, n=12; males, n=17).

**Results:**

After correction for multiple testing, we observed that circulating levels of 11-deoxycortisol, 17α-hydroxyprogesterone in females, and progesterone in males were significantly different between HC, ME/CFSmm, and ME/CFSsa. Comparing two independent groups, we found that female ME/CFSsa had higher levels of 11-deoxycortisol (vs. HC and ME/CFSmm) and 17α-hydroxyprogesterone (vs. HC). In addition, female ME/CFSmm showed a significant increase in progesterone levels compared to HC. In contrast, our study found that male ME/CFSmm had lower circulating levels of cortisol and corticosterone, while progesterone levels were elevated compared to HC. In addition to these univariate analyses, our correlational and multivariate approaches identified differential associations between our study groups. Also, using two-component partial least squares discriminant analysis (PLS-DA), we were able to discriminate ME/CFS from HC with an accuracy of 0.712 and 0.846 for females and males, respectively.

**Conclusion:**

Our findings suggest the potential value of including steroid hormones in future studies aimed at improving stratification in ME/CFS. Additionally, our results provide new perspectives to explore the clinical relevance of these differences within specific patient subgroups.

**Graphical abstract:**

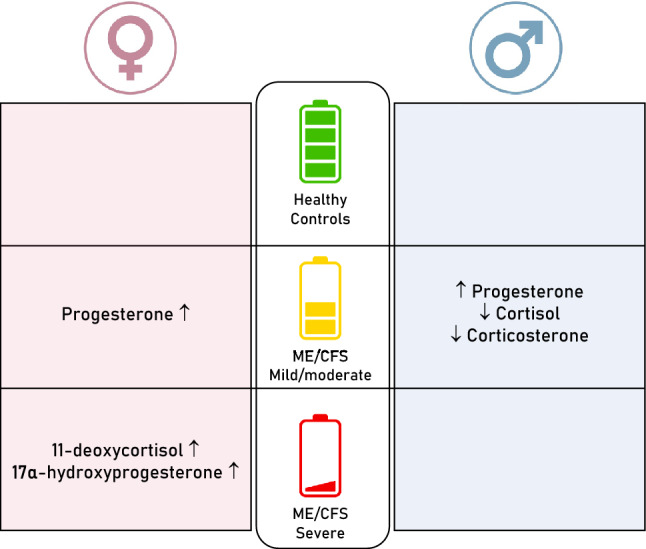

**Supplementary Information:**

The online version contains supplementary material available at 10.1007/s40618-024-02334-1.

## Introduction

Myalgic encephalomyelitis/chronic fatigue syndrome (ME/CFS) is a debilitating multisystem disease that primarily manifests as the inability to perform or participate in routine activities that were possible before the onset of the disease [1]. The decrease in activity level is accompanied by core symptoms, such as unrefreshing sleep and fatigue at rest, which persists for more than six months. Post-exertional malaise (PEM), defined as the worsening of symptoms after minimal physical or mental effort, is widely accepted as the hallmark feature for diagnosing ME/CFS [1, 2]. The clinical manifestation of ME/CFS varies from a mild form, in which patients are still able to participate in social life (e.g., work, school), to moderate and severe forms, characterized by individuals being primarily homebound and bedridden. The most extreme manifestation of the disease occurs in people who are completely dependent on assistance for basic daily needs, such as eating and repositioning in bed [1, 3]. As a result, ME/CFS not only negatively impacts the quality of life of individuals and caregivers but also places a substantial and often silent burden on the healthcare system. This is due to affected individuals often remaining undiagnosed due to a lack of standardized clinical assessment and diagnostic markers [2]. Although the etiology remains elusive, up to 75% of ME/CFS cases report an infectious episode preceding the onset of the disease [1, 2]. Current evidence strongly suggests that ME/CFS is associated with immune, metabolic, and vascular abnormalities [4]. However, despite the higher prevalence in women than in men [1], endocrinological studies focusing on the steroid hormones that may underlie this sex bias are scarce.

Steroid hormones are synthesized from cholesterol primarily in the adrenal gland, gonads, and placenta, playing crucial regulatory roles in various physiological and pathological processes [5–7]. The adrenal gland produces mineralocorticoids to regulate blood volume and pressure, and glucocorticoids to modulate metabolism and the immune system [8, 9]. The gonads synthesize sex steroid hormones in both females and males. These hormones not only modulate sexual function but also immune responses, which can influence sex-specific susceptibility to infectious and autoimmune diseases [10]. Previous studies have reported differences in steroid hormones levels between ME/CFS patients and healthy controls (HC) using various experimental approaches [11–16]. However, there has been a lack of research aimed at analyzing potential differential patterns based on sex and disease severity. Therefore, our experimental design focused on measuring the circulating levels of nine steroid hormones using highly specific quantitative measurements by ultra-high performance liquid chromatography coupled to triple quadrupole tandem mass spectrometry (UHPLC-MS/MS). These nine steroid hormones are crucial in biosynthetic and metabolic pathways related to steroidogenesis [5]. The set of steroid hormones was classified into four categories: mineralocorticoids (aldosterone), glucocorticoids (cortisol, corticosterone, 11-deoxycortisol, cortisone), androgens (androstenedione, testosterone), and progestins (progesterone, 17α-hydroxyprogesterone). Plasma samples from female and male ME/CFS patients with mild/moderate (ME/CFSmm) and severe (ME/CFSsa) symptoms were compared to their respective healthy controls (HC).

## Materials and methods

### Plasma samples and clinical data

This retrospective study utilized plasma samples collected before 10am from both female and male individuals provided by the UK ME/CFS Biobank (UKMEB) [17, 18]. The female cohort comprised 56 individuals, categorized into HC (*n* = 12; 21%), ME/CFSmm (*n* = 20; 36%), and ME/CFSsa patients (*n* = 24; 43%). The male cohort included 31 individuals, distributed into HC (*n* = 17; 55%), ME/CFSmm (*n* = 8; 26%), and ME/CFSsa patients (*n* = 6; 19%) (Table 1).

**Table 1:** Demographics of the female and male cohorts including continuous variables, clinical assessments, blood tests, and SF-36 questionnaire

Female	HC		ME/CFSmm		ME/CFSsa		*p* value
	(*n*) 12	(%) 21	(*n*) 20	(%) 36	(*n*) 24	(%) 43	
	Median	IQR	Median	IQR	Median	IQR	
*Continuous variables*							
Age (years)	38	28–45	43	30–48	46	31–53	0.2174
Years of disease ^§^	-	-	5	2–12	11	5–21	0.0301*
Fatigue severity scale	16	12–22	60	53–61	60	56–62	< 0.0001****
*Clinical assessments*							
Waist circumference (cm)	76	73–104	89	82–97	76	69–85	0.0150*
Pain analog scale	0.0	0.0–1.1	2.4	1.6–3.4	4.5	1.9–6.9	0.0002***
Fatigue analog scale	0.8	0.1–2.5	4.8	3.1–7.0	7.4	6.0–8.5	< 0.0001****
*Blood tests*							
Inorganic phosphate (mM)	0.9	0.8–1.0	1.1	0.9–1.6	1.1	0.9–1.1.2	0.0394*
Bilirubin (µM)	11	6–11	6	3–8	6	5–7	0.0082**
Creatine phosphokinase (U/L)	83	75–233	67	56–100	59	47–73	0.0038**
Vitamin B12	380	197–488	350	259–501	521	355–689	0.0330*
*SF-36 questionnaire*							
Physical functioning	58	58–58	32	29–40	21	19–27	< 0.0001****
Role physical	57	53–57	30	22–35	21	21–26	< 0.0001****
Bodily pain	56	52–60	40	34–46	34	27–46	< 0.0001****
General health	61	58–63	31	26–33	26	24–31	< 0.0001****
Vitality	53	47–59	29	26–35	26	23–32	< 0.0001****
Social functioning	57	54–57	27	18–32	17	17–22	< 0.0001****
Role emotional	56	56–56	42	33–52	47	35–56	0.0070**
Mental health	52	46–58	40	36–48	48	38–53	0.0165*
Physical component summary	58	55–59	30	27–34	20	17–27	< 0.0001****
Mental health component summary	54	48–57	41	32–45	46	36–49	0.0032**

Male	HC		ME/CFSmm		ME/CFSsa		*p* value
	(*n*) 17	(%) 55	(*n*) 8	(%) 26	(*n*) 6	(%) 19	
	Median	IQR	Median	IQR	Median	IQR	
*Continuous variables*							
Age (years)	33	20–47	40	36–49	35	34–40	0.3181
Years of disease ^§^	–	–	4	1–9	9	3–23	0.3906
Fatigue severity scale	2.0	1.6–2.4	6.6	5.7–6.7	6.4	6.1–6.8	< 0.0001****
*Clinical assessments*							
Body fat	16	9–22	21	15–25	27	20–38	0.0385*
Pain analog scale	0.3	0.0–1.4	2.9	1.2–5.6	3.6	1.9–6.0	0.0017**
Fatigue analog scale	0.8	0.0–1.6	5.9	3.3–7.2	6.3	4.3–7.8	0.0003***
*Blood tests*							
Glomerular filtration rate (mL/min)	84	81–90	90	86–90	77	63–90	0.0464*
*SF-36 questionnaire*							
Physical functioning	58	56–58	35	29–36	21	21–24	< 0.0001****
Role physical	57	55–57	23	21–28	21	21–27	< 0.0001****
Bodily pain	56	56–62	42	31–47	38	27–46	< 0.0003***
General health	59	53–66	33	28–36	30	27–33	< 0.0001****
Vitality	53	50–61	26	23–32	27	23–39	< 0.0001****
Social functioning	57	57–57	22	17–27	17	17–29	< 0.0001****
Mental health	56	49–59	40	35–51	50	48–57	0.0350*
Physical component summary	59	55–60	28	25–35	20	13–21	< 0.0001****

The study population is described based on sex, age, years of disease, fatigue severity scale scores, clinical assessments, blood tests, and SF-36 questionnaire, with values reported as median and interquartile range (IQR). Participants were categorized into three groups: HC (healthy controls), ME/CFSmm (mild/moderate ME/CFS patients), and ME/CFSsa (severely affected ME/CFS patients). Statistical significance was determined using one-way ANOVA for normally distributed parameters or Kruskal–Wallis test for non-normally distributed parameters. The median and IQR for the variable *years of disease* (§) were calculated by comparing two independent groups (ME/CFSmm vs ME/CFSsa) using the non-parametric Mann–Whitney t-test

Individuals diagnosed with ME/CFS were evaluated by a clinician based on the Canadian Consensus [19] and/or CDC-1994 (‘Fukuda’) [20] criteria. The diagnosis was validated through responses on the Symptoms Assessment form, ensuring compliance with the case definition and study eligibility. Participants in the study completed a set of questionnaires aimed at evaluating disability levels, including the Fatigue Severity Scale, measuring the severity of fatigue symptoms to provide insights into their impact. Additionally, the Pain and Fatigue Analog Scale assessed the subjective experience of pain and fatigue on a visual analog scale, allowing participants to express the intensity of their symptoms. The Medical Outcomes Survey Short Form (SF-36v2) was employed as a comprehensive multidimensional instrument to evaluate various aspects of participants' physical and mental well-being, offering a holistic view of health-related quality of life. These instruments collectively contributed to a thorough assessment of participants' health status and functional components [21, 22].

Exclusion criteria for participants included individuals who, within the preceding three months, (1) had used drugs known to modify immune function (e.g., azathioprine, cyclosporine, methotrexate, steroids) or had taken antiviral medications; (2) had received any vaccinations; (3) had a history of acute or chronic infectious diseases such as hepatitis B and C, tuberculosis, or HIV (excluding infections by herpes virus or other retroviruses); (4) had another severe medical condition such as cancer, coronary heart disease, or uncontrolled diabetes; (5) had a severe mood disorder; (6) had been pregnant or breastfeeding in the prior 12 months; or (7) presented with morbid obesity (BMI ≥ 40). Home visits were conducted to recruit patients with mobility restrictions (severely affected), while healthy subjects and mild/moderate patients were invited to a recruiting center for clinical assessment and blood sampling [21, 22].

### Standards and solvents

Certified reference standards of corticosterone solution (C-117-1ML; CAS: 50-22-6), cortisol solution (C-106-1ML; CAS: 50-23-7), cortisone solution (C-130-1ML; CAS: 53-06-5), and 11-deoxycortisol solution (D-061-1ML; CAS: 152-58-9) were purchased from Merck (KgaA, Darmstadt, Germany). Stable isotope-labeled standards, namely aldosterone-D_7_ (CAS: 1261254-31-2), androstene-3,17-dione-^13^C_3_ (CAS: 327048-86-2), corticosterone-D_4_ (CAS: 2243253-91-8), cortisol-D_4_ (CAS: 73565-87-4), cortisone-^13^C_3_ (CAS: 2350278-95-2), 11-deoxycortisol-D_5_ (CAS: 1258063-56-7), 17α-hydroxyprogesterone-D_8_ (CAS: 850023-80-2), progesterone-D_9_ (CAS: 15775-74-3), and testosterone-^13^C_3_ (CAS: 327048-83-9) were also obtained from Merck (KgaA, Darmstadt, Germany). Purified water was prepared in-house using a Milli-Q water system from Millipore (Bedford, MA, USA). HPLC-grade methanol (MeOH; 34885-1L-M), ethyl acetate (EtOAc; 1.00868), methyl-tert-butyl ether (MTBE; 34875-1L-M), LC–MS-grade acetonitrile (ACN; 1.00029. 1000), 2-propanol (IPA; 1.02781.1000), formic acid (FA; 1002531000), and ammonium fluoride (338869-25G) were purchased from Merck (KGaA, Darmstadt, Germany).

### Preparation of standard solutions

To prepare the stock solution of the internal standard (IS), the stable isotope-labeled compounds were combined in ACN to achieve final concentrations of 2000 ng/mL for aldosterone-D_7_, cortisone-^13^C_3_ and cortisol-D_4_, 400 ng/mL for corticosterone-D_4_ and 11-deoxycortisol-D_5_, 200 ng/mL androstene-3,17-dione-^13^C_3_, testosterone-^13^C_3_, 17α -hydroxyprogesterone-D_8_, and 100 ng/mL for progesterone-D_9_. The working solution (WSL) was obtained by diluting the stock 1:10 in MeOH. The standard stock solution mixture in MeOH with a concentration of 1000 ng/mL was prepared using the nine individual steroid hormones. A 500 μL aliquot of the stock solution mixture was transferred to a 5 mL volumetric flask and brought up to the highest calibration point with a 50:50 (v:v) mixture of MeOH and H_2_O. Nineteen additional dilutions were made using a 50:50 (v:v) MeOH:H_2_O mixture, and the calibration curve samples were prepared by adding 10 μL of the IS WSL solution to 100 μL of the mixed standard solution to cover a calibration range from 0.00019 ng/mL to 100 ng/mL. Stock solutions were stored at –20°C and allowed to reach room temperature before use.

### Sample preparation

Supported liquid extraction (SLE) was employed to mitigate the impact of the sample matrix. The SLEs were purchased from Agilent Technologies (5610-2005). Briefly, 100 µL of plasma were combined with 100 µL of H_2_O and 10 µL of IS WSL. The resultant mixture was transferred to the SLE tube, placed onto the sorbent bed with gentle pressure (2–3 psi), and allowed to equilibrate for 5 min. Subsequently, 400 µL of a 1:1 mixture of methyl-tert-butyl ether (MTBE) and ethyl acetate (EtOAc) were introduced into each tube, followed by elution at a rate of 1 drop per second (2 psi). This elution process was iterated three times, and a final application of 6 psi was employed to desiccate the sorbent. The entire eluent was dried with nitrogen (N_2_) flow at 40°C in a TurboVap water bath (Biotage, Sweden) and then reconstituted with 100 µL of MeOH. Samples were stored at 4 °C overnight, subjected to centrifugation at 2500×*g* for 5 min, and subsequently loaded into the autosampler.

### UHPLC-MS/MS measurements and conditions

All separations were performed on a 1290 Infinity UHPLC system (Agilent Technologies) equipped with an Agilent ZORBAX RRHD Eclipse Plus C_18_ column (2.1 × 100 mm; 1.8 µm; 821725-902) and a ZORBAX RRHD C_18_ guard column (2.1 × 5 mm; 1.8 µm; 821725-901). The mobile phases comprised 0.2 mM ammonium fluoride in H_2_O as mobile phase A and 0.2 mM ammonium fluoride in MeOH as mobile phase B. The gradient conditions were as follows: 0–3.0 min; 50–60% B; 3.0–7.0 min, 60–86% B; 7.0–7.1 min; 86-100% B, followed by a return to the initial conditions. The total chromatographic run time was 8.5 min. The flow rate was set to 0.4 mL/min, and the column temperature was maintained at 40 °C. The injection volume was 3 µL, and a needle wash with 1:1:1:1 ACN/MeOH/IPA/H_2_O with 0.2% FA was utilized. Mass detection was carried out in dynamic multiple reaction monitoring (dMRM) mode on an Agilent 6460 triple quadrupole system using positive electrospray ionization (ESI) mode. Specific settings can be found in Tables S1 and S2, while Figure S1 provides a representative UHPLC-dMRM chromatogram.

### Statistical analysis

For comparing the means of clinical variables among the three groups (HC, ME/CFSmm, and ME/CFSsa), one-way ANOVA was employed when the respective data followed a normal distribution. In cases where normal distribution was not met, the non-parametric Kruskal–Wallis test was used. The significance level for these tests was set at 5%. Central tendency and variability in the dataset were estimated using the median and interquartile range (IQR).

To assess differences in the circulating levels of steroid hormones in female and male ME/CFS patients compared to HC, ANOVA, and Kruskal–Wallis tests were utilized to determine statistical significance. Unadjusted *p* values were computed for each test. To address multiple testing, we controlled the false discovery rate (FDR) at a 5% level using the Benjamini–Hochberg (BH) and Benjamini–Yekutieli (BY) procedures.

We also conducted a predictive analysis to assess the disease status (HC versus ME/CFS) of each participant using the following classifiers and the nine steroid hormones as respective predictors: (i) linear discriminant analysis (LDA), (ii) random forest (RF), and (iii) partial least square discriminant analysis (PLS-DA). This analysis was carried out separately for male and female datasets. In PLS-DA, the analysis was performed with one and two latent components. Using a higher number of components in PLS-DA, while potentially enhancing predictive performance, was prone to overfitting the data. For LDA and PLS-DA, the probability of an individual being an ME/CFS patient was estimated based on a leave-one-out procedure. For RF, the same probability was estimated using Bootstrap with 10,000 simulated decision trees.

After obtaining the classification probability for each participant, we constructed a Receiver Operating Characteristic (ROC) curve, where 1-specificity (x-axis) was plotted against sensitivity (y-axis). The corresponding area under the curve (AUC) and the point on the curve closest to the pair (0,1) (representing perfect classification) were determined. This point served as the optimal cutoff to predict the health status of each individual. Finally, we utilized the predicted health status to estimate accuracy (proportion of individuals with correctly predicted health status), sensitivity (Se, proportion of cases with correctly predicted health status), and specificity (Sp, proportion of controls with correctly predicted health status) associated with each classifier. This analysis was carried out in the R software using the following packages: *MASS* (for LDA) [23], *randomForest* (for RF) [24], *caret* (for PLS-DA) [25], *pROC* (for ROC curve and AUC calculation) [26], *OptimalCutpoints* (for optimal cutoff estimation, accuracy, sensitivity, and specificity) [27].

Analyses utilizing Spearman's correlation coefficient (*R*_sp_) were conducted to examine the statistical relationship between data from two steroid hormones. Each steroid hormone was ranked from lowest to highest, and the correlation coefficient along with corresponding *p* values were computed using *GraphPad Prism*. Next, to compare Spearman's correlation matrices between HC and individuals with ME/CFS (or its subgroups), with or without the inclusion of data on aldosterone (which contains missing data), a permutation variant of Jennrich's test was implemented. This test was originally designed for comparing two Pearson's correlation matrices [28]. The computational procedure involved the following steps: (i) fixing the values of the group variable; (ii) permutating the original data set; (iii) computing the Jennrich's test statistic in the permutated data set; (iv) repeating steps (ii) and (iii) until obtaining 1000 values of the test statistic; (v) estimating the *p* value by determining the proportion of times the observed test statistic in the original dataset was higher than the values of the test statistic based on the permutated datasets.

Principal component analysis (PCA) of the steroid hormones data was employed to evaluate the similarity of study participants concerning sex and disease severity. In this analysis, we utilized the first and second principal components, as these components explained over 90% of the data variability. Heatmaps were generated for the entire dataset and separately for the female and male cohorts using the *MetaboAnalyst* R package [29] to conduct a thorough analysis of the data. Clustering of the data was performed using Euclidean distance, aiming to unveil underlying patterns and relationships. To identify distinct subgroups, the resulting clusters were further segmented into multiple groups. Data visualization was executed using the *pheatmap* R package [30].

## Results

### Baseline demographics

Table 1 provides an overview of the demographic and clinical characteristics of the female and male participants. Noteworthy clinical differences were primarily identified in waist circumference for the female cohort and body fat for the male cohort when comparing the three independent groups. Applying the same statistical criteria, median values of blood-related tests, such as inorganic phosphate, bilirubin, creatine phosphokinase, and vitamin B_12_ in the female cohort, and glomerular filtration rate in the male cohort, showed differences among the three groups. Further distinctions were identified in the female cohort when comparing the aforementioned parameters between two independent groups using the non-parametric Mann–Whitney test. For instance, statistically significant differences were observed in body mass index (ME/CFSmm vs. ME/CFSsa; *p* = 0.0238), body muscle (HC vs. ME/CFSsa; *p* = 0.0249), and C-reactive protein (HC vs. ME/CFSsa; *p* = 0.0161). Conversely, pulse oximetry (HC vs. ME/CFSmm; *p* = 0.0226), albumin (HC vs. ME/CFSmm; *p* = 0.0135), and thyroid stimulating hormone (HC vs. ME/CFSmm; *p* = 0.0439) demonstrated differences in the male cohort using the same criteria outlined above. Moreover, the median scores for various functional components, as assessed by the questionnaires Fatigue Severity Scale, Pain and Fatigue Analog Scale, and SF-36, revealed significant reductions in both female and male subgroups of ME/CFS patients when compared with their respective healthy counterparts (Table 1).

**Table 2:** Plasma levels of steroid hormones in female and male ME/CFS patients compared to healthy controls

Female	HC		ME/CFSmm		ME/CFSsa	
	(*n*) 12	(%) 21	(*n*) 20	(%) 36	(*n*) 24	(%) 43
Steroid hormones (ng/mL)	Median	IQR	Median	IQR	Median	IQR
Cortisone	21.02	17.25–25.17	19.77	15.62–21.91	19.57	17.79–23.00
Cortisol	94.55	84.31–138.70	89.84	68.87–147.40	110.70	76.61–131.40
Corticosterone	1.23	1.02–1.87	1.12	0.85–3.12	1.97	0.86–4.06
11-deoxycortisol	0.12	0.08–0.25	0.13	0.09–0.18	0.22	0.14–0.56
Aldosterone	0.10	0.06–0.16	0.11	0.08–0.30	0.08	0.05–0.13
Androstenedione	0.78	0.54–1.11	0.65	0.43–0.79	0.80	0.57–1.14
Testosterone	0.23	0.13–0.30	0.20	0.15–0.26	0.23	0.16–0.32
17α-hydroxyprogesterone	0.23	0.14–0.37	0.56	0.20–0.67	1.24	0.26–1.66
Progesterone	0.09	0.06–0.15	0.69	0.19–3.79	0.36	0.08–1.10

Male	HC		ME/CFSmm		ME/CFSsa	
	(*n*) 17	(%) 55	(*n*) 8	(%) 26	(*n*) 6	(%) 19
Steroid hormones (ng/mL)	Median	IQR	Median	IQR	Median	IQR
Cortisone	20.67	17.22–25.17	19.48	15.29–26.10	20.24	13.62–25.01
Cortisol	120.80	94.11–134.30	70.03	55.18–119.80	99.32	49.76–153.50
Corticosterone	1.89	1.12–3.43	0.67	0.47–2.27	1.47	0.79–10.37
11-deoxycortisol	0.16	0.12–0.26	0.13	0.08–0.24	0.23	0.11–0.84
Aldosterone	0.07	0.05–0.16	0.11	0.05–0.17	0.07	0.05–0.21
Androstenedione	0.74	0.59–0.86	0.54	0.42–0.83	0.65	0.52–0.96
Testosterone	4.78	3.07–6.75	4.47	2.81–5.13	3.32	2.57–4.89
17α-hydroxyprogesterone	0.64	0.38–0.83	1.02	0.65–1.46	0.55	0.43–1.14
Progesterone	0.07	0.04–0.08	0.48	0.12–0.71	0.07	0.06–0.23

Circulating levels of cortisone, cortisol, corticosterone, 11-deoxycortisol, aldosterone, androstenedione, testosterone, 17α-hydroxyprogesterone, and progesterone were quantified using UHPLC-MS/MS in plasma samples from healthy controls (HC), mild/moderate (ME/CFSmm), and severely affected (ME/CFSsa) female and male individuals. The table presents the number of samples (*n*), percentage (%), median, and interquartile range (IQR) values

### Univariate analysis of steroid hormone data

**Fig. 1:**
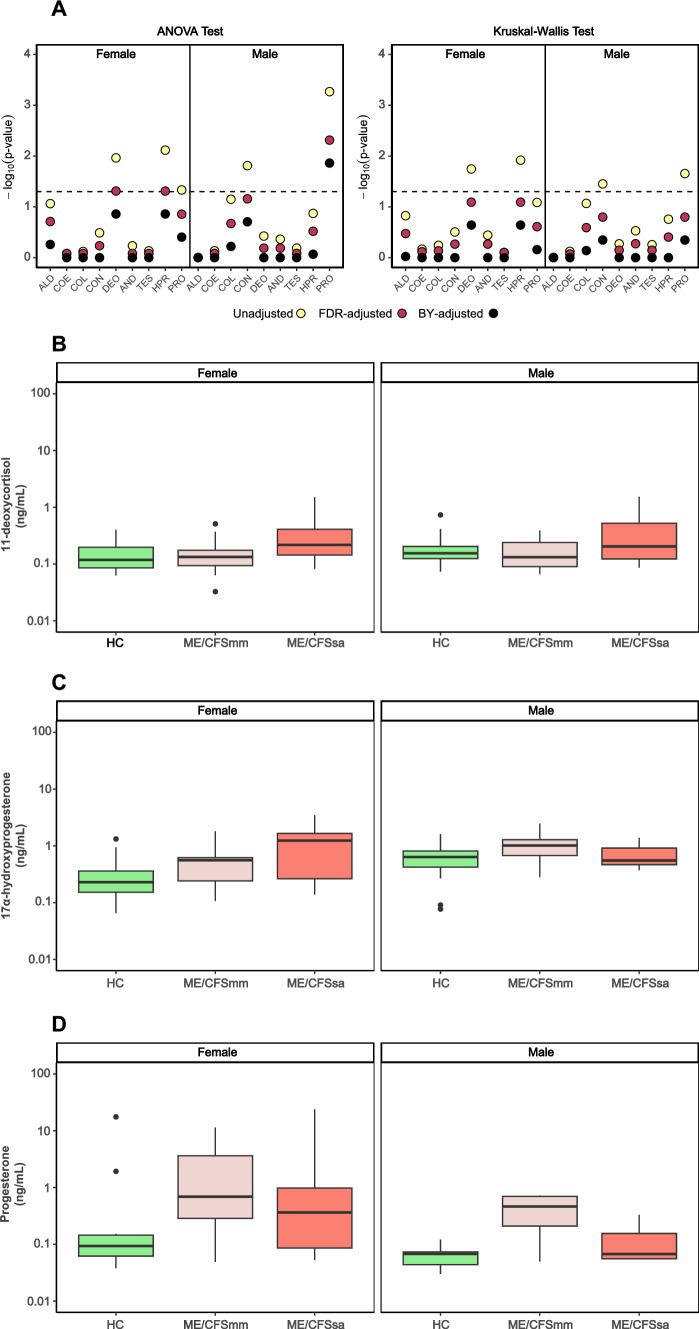
Plasma levels of steroid hormones in female and male ME/CFS patients compared to healthy controls. The concentration of a steroid hormone panel, including cortisone (COE), cortisol (COL), corticosterone (CON), 11-deoxycortisol (DEO), aldosterone (ALD), androstenedione (AND), testosterone (TES), 17α-hydroxyprogesterone (HPR), and progesterone (PRO) was assessed using UHPLC-MS/MS in plasma samples from cohorts of female and male ME/CFS patients with mild/moderate (ME/CFSmm) and severe (ME/CFSsa) presentations, in comparison to their respective healthy controls (HC). (A) ANOVA and Kruskal–Wallis tests were employed to determine *p* values. For each test, unadjusted (yellow circles), false discovery rate (FDR)-adjusted (light red circles), and Benjamini–Yekutieli (BY)-adjusted (black circles) *p* values were calculated. Each circle represents the − log_10_(*p* value) of a specific statistical association test, while the dashed line represents − log_10_(0.05), above which it was considered a statistically significant association. Benjamini–Hochberg *p* values were adjusted for 11-deoxycortisol (**B**), 17-hydroxyprogesterone (**C**), and progesterone (**D**)

The concentrations of a panel of steroid hormones, including cortisone, cortisol, corticosterone, 11-deoxycortisol, aldosterone, androstenedione, testosterone, 17α-hydroxyprogesterone, and progesterone, were measured in plasma samples by UHPLC-MS/MS (Table 2). We employed ANOVA and Kruskal–Wallis tests to assess differences in steroid hormone levels between female and male ME/CFS patients compared to HC (Fig. 1A). For each test, unadjusted, BH-adjusted, and BY-adjusted *p* values were calculated (Table S3). Following correction for multiple testing, only circulating levels of 11-deoxycortisol (*p* = 0.049) and 17α-hydroxyprogesterone (*p* = 0.049) in females and progesterone (*p* = 0.005) in males showed significant differences among our three groups (Fig. 1B–D; Table S3). Additionally, the BH procedure was utilized to determine the statistical significance of potential differences when comparing two independent groups (Table S4). In our female cohort, ME/CFSsa exhibited elevated circulating levels of 11-deoxycortisol compared to both HC (*p* = 0.0276) and ME/CFSmm (*p* = 0.0269), as well as higher 17α-hydroxyprogesterone versus HC (*p* = 0.0129). Furthermore, plasma progesterone levels were higher in ME/CFSmm than in HC (*p* = 0.0406). Using the same statistical approach in our male cohort, ME/CFSmm displayed lower circulating levels of cortisol (*p* = 0.0260) and corticosterone (*p* = 0.0109) compared to HC. Conversely, ME/CFSmm exhibited higher progesterone levels than HC (*p* = 0.0004). No statistical differences were found in the set of measured steroid hormones in the male cohort when comparing HC versus ME/CFSsa and ME/CFSmm versus ME/CFSsa (Table S4).

### Correlation analysis of steroid hormone data

**Fig. 2:**
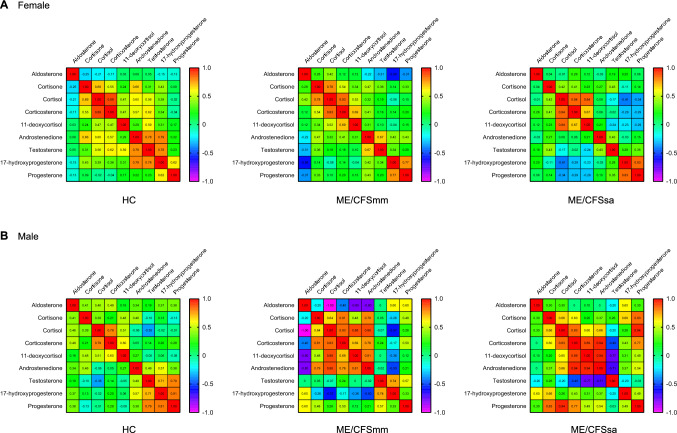
Degree of association between steroid hormones in plasma from female and male healthy controls, mild/moderate and severe ME/CFS patients. The degree of association among steroid hormones (cortisone, cortisol, corticosterone, 11-deoxycortisol, aldosterone, androstenedione, testosterone, 17α-hydroxyprogesterone, and progesterone) in female (**A**) and male (**B**) individuals was assessed using the nonparametric Spearman's test. Both female and male cohorts were categorized into: HC, representing participants recruited as healthy controls; ME/CFSmm, denoting participants recruited as mild/moderate ME/CFS patients; and ME/CFSsa, indicating participants recruited as severe ME/CFS patients. Correlation coefficients were classified as very high, high, moderate, low, and little or no correlation, with magnitudes ranging from 0.9 to 1.0, 0.7–0.9, 0.5–0.7, 0.3–0.5, and < 0.3, respectively. A positive Spearman's rank correlation coefficient indicates a direct association, while a negative coefficient indicates an inverse association

We performed non-parametric Spearman's correlation coefficient (*R*_sp_) analysis to evaluate the degree of association between specific steroid hormones in both the female and male cohorts (Fig. 2). In the female HC group, we identified eight significant positive correlations: cortisol versus corticosterone (*R*_sp_ = 0.89, *p* = 0.003); androstenedione versus 17-hydroxyprogesterone (*R*_sp_ = 0.79, *p* = 0.003); testosterone versus androstenedione (*R*_sp_ = 0.78, *p* = 0.004); testosterone versus 17-hydroxyprogesterone (*R*_sp_ = 0.78, *p* = 0.004); cortisol versus cortisone (*R*_sp_ = 0.69, *p* = 0.017); androstenedione versus cortisone (*R*_sp_ = 0.66, *p* = 0.022); testosterone versus corticosterone (*R*_sp_ = 0.62, *p* = 0.037); progesterone versus 17-hydroxyprogesterone (*R*_sp_ = 0.62, *p* = 0.037). Comparing the female HC to ME/CFSmm, five of the eight positive associations were also found: cortisol versus corticosterone (*R*_sp_ = 0.83, *p* = 0.001); cortisol versus cortisone (*R*_sp_ = 0.78, *p* = 0.001); progesterone versus 17-hydroxyprogesterone (*R*_sp_ = 0.77, *p* = 0.001); testosterone versus androstenedione (*R*_sp_ = 0.67, *p* = 0.001); androstenedione versus cortisone (*R*_sp_ = 0.47, *p* = 0.036). Additionally, three positive significant correlations were observed for corticosterone versus 11-deoxycortisol (*R*_sp_ = 0.69, *p* = 0.001); corticosterone versus cortisone (*R*_sp_ = 0.54, *p* = 0.013); cortisol versus 11-deoxycortisol (*R*_sp_ = 0.52, *p* = 0.021); and one negative correlation for 17-hydroxyprogesterone versus aldosterone (*R*_sp_ = -0.58, *p* = 0.016). In the female ME/CFSsa group, only 4 positive significant correlations were identified, with two overlapping with the HC group: cortisol versus corticosterone (*R*_sp_ = 0.84, *p* = 0.001); progesterone versus 17-hydroxyprogesterone (*R*_sp_ = 0.83, *p* = 0.001) and two overlapping with the ME/CFSmm group: cortisol versus 11-deoxycortisol (*R*_sp_ = 0.84, *p* = 0.001); corticosterone versus 11-deoxycortisol (*R*_sp_ = 0.67, *p* = 0.001) (Fig. 2A).

On the contrary, we identified six significantly positive significant correlations in our male HC group: progesterone versus 17-hydroxyprogesterone (*R*_sp_ = 0.81, *p* = 0.001); cortisol versus corticosterone (*R*_sp_ = 0.79, *p* = 0.003); testosterone versus progesterone (*R*_sp_ = 0.79, *p* = 0.001); testosterone versus 17-hydroxyprogesterone (*R*_sp_ = 0.71, *p* = 0.002); corticosterone versus 11-deoxycortisol (*R*_sp_ = 0.60, *p* = 0.012); androstenedione versus 17-hydroxyprogesterone (*R*_sp_ = 0.57, *p* = 0.018). Compared to the male HC group, only one of these six positive associations was found in the male ME/CFSmm counterparts: cortisol versus corticosterone (*R*_sp_ = 0.83, *p* = 0.015). However, we additionally observed six significantly positive correlations for cortisol versus 11-deoxycortisol (*R*_sp_ = 0.88, *p* = 0.007); androstenedione versus cortisol (*R*_sp_ = 0.88, *p* = 0.007); corticosterone versus cortisone (*R*_sp_ = 0.81, *p* = 0.022); androstenedione versus 11-deoxycortisol (*R*_sp_ = 0.81, *p* = 0.022); androstenedione versus cortisone (*R*_sp_ = 0.79, *p* = 0.028); androstenedione versus corticosterone (*R*_sp_ = 0.76, *p* = 0.037). Finally, we identified four significantly positive correlations in male ME/CFSsa, one of which was also found in HC: corticosterone versus 11-deoxycortisol (*R*_sp_ = 0.89, *p* = 0.033), and two in ME/CFSmm: androstenedione versus corticosterone (*R*_sp_ = 0.94, *p* = 0.017); androstenedione versus 11-deoxycortisol (*R*_sp_ = 0.94, *p* = 0.017). Interestingly, a positive significant association was observed exclusively in male ME/CFSsa compared to either ME/CFSmm or HC, progesterone versus cortisol (*R*_sp_ = 0.94, *p* = 0.017) (Fig. 2B). To complement our analysis, we also calculated *R*_sp_-based correlation matrices for the nine measured steroid hormones in each study group. Our results showed no statistical difference between different pairs of correlation matrices associated with the study groups (Figure S2; Table S5).

### Multivariate analysis of steroid hormone data

We conducted principal component analysis (PCA) to identify potential clusters within our study groups (Figure S3). This analysis aimed to visualize our cohort of individuals based on steroid hormones, sex, and disease severity in a two-dimensional plot. Using the first two principal components did not reveal any distinct clusters based on severity, sex, or a combination of both (Figure S3A). However, plotting the second and third principal components allowed us to differentiate between female and male individuals, though not individuals with varying disease severity (Figure S3B). Consequently, the steroid hormone data seemed to provide limited information about the study groups.

We further employed various statistical approaches, including linear discriminant analysis (LDA), random forest (RF), and partial least squares discriminant analysis (PLS-DA), to assess the predictive and discriminative capacities of the nine steroid hormones in distinguishing between HC and ME/CFS (Figure S4; Table 3). The two-component PLD-DA demonstrated the best predictive performance for the female cohort, with an estimated AUC of 0.712 and accuracy of 0.702. Notably, this classifier performed well in both cases and controls (Sensitivity = 0.714 vs. Specificity = 0.667). Concerning the male cohort, three out of four classifiers achieved the same level of predictive accuracy (0.818). However, the two-component PLD-DA emerged as the best classifier based on AUC. Similar to the female cohort, this classifier achieved a reasonable balance between sensitivity and specificity (0.778 vs. 0.846, respectively).

**Table 3:** Predictive analysis using the nine measured steroid hormones as respective predictors to estimate the health status of each participant

Statistical method	HC versus ME/CFS			
	AUC	Accuracy	Sensitivity	Specificity
Female				
LDA	0.602	0.681	0.714	0.583
RF	0.571	0.574	0.514	0.750
PLS-DA (One component)	0.519	0.553	0.514	0.667
PLS-DA (Two components)	0.712	0.702	0.714	0.667
Male				
LDA	0.658	0.727	0.667	0.769
RF	0.641	0.818	0.556	1.000
PLS-DA (One component)	0.735	0.818	0.667	0.923
PLS-DA (Two components)	0.846	0.818	0.778	0.846

The datasets from our cohorts of female and male individuals were subjected to separate analysis to separate analyses employing linear discriminant analysis (LDA), random forest (RF), and partial least squares discriminant analysis (PLS-DA). The predictive accuracy was assessed through the area under the receiver operating characteristic curve (AUC)

**Fig. 3:**
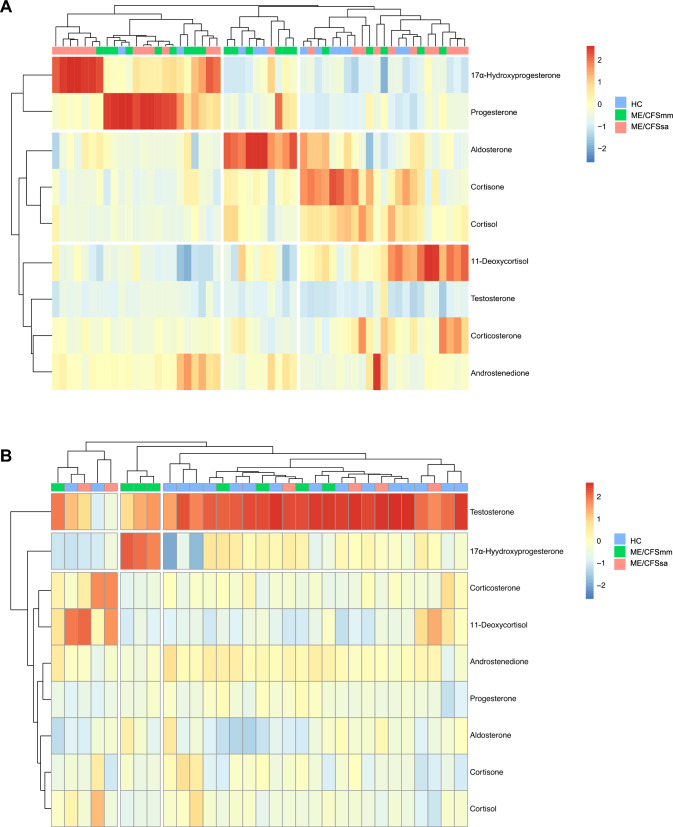
Hierarchical clustering analysis based on plasma steroid hormone levels in female and male cohorts. Data obtained from cortisone, cortisol, corticosterone, 11-deoxycortisol, aldosterone, androstenedione, testosterone, 17α-hydroxyprogesterone, and progesterone were subjected to hierarchical clustering analysis utilizing the Euclidean distance metric. The results were visualized through heatmaps in both the female (**A**) and male (**B**) cohorts

To complement our findings, we conducted a clustering analysis to categorize our study cohorts based on the similarity of their circulating steroid hormone profiles. Within the female cohort, three clusters were observed primarily driven by the concentrations of 17α-hydroxyprogesterone, progesterone, and 11-deoxycortisol in ME/CFS patients, with no clear differentiation between subgroups. While two additional patterns emerged related to aldosterone and cortisone, individuals from HC, ME/CFSmm, and ME/CFSsa were clustered together (Fig. 3A). The same analysis was applied to the male dataset, and despite a prominent cluster related to testosterone, no differences were observed between our study groups (Fig. 3B).

## Discussion

Despite evidence indicating a higher prevalence of ME/CFS in women compared to men (3:1 ratio) [1], there remains a significant research gap concerning potential differences in circulating levels of steroid hormones in patients stratified by both sex and disease severity. Steroid hormones are synthesized by both males and females, displaying variations in circulating levels and effects on target tissues [5–9]. While testosterone is predominantly produced in males, and estrogen and progesterone are primarily synthesized in females [31], these differences have historically limited research on the role of testosterone in women and estrogen and progesterone in men [32]. Notably, our study revealed elevated progesterone levels in both female and male ME/CFSmm patients compared to their healthy counterparts. While the biological effects of progesterone in men are presently not fully understood, there is evidence suggesting an immunosuppressive effect of progesterone in women [33]. Our findings indicate that basal plasma progesterone is elevated in male ME/CFSmm patients, similar to previous reports in female patients [34], proving an interesting avenue for further investigation. Importantly, progesterone has been associated with a hypercoagulable state [35]. This is particularly noteworthy considering recent reports of fibrinoid microclots in ME/CFS patients [36]. However, additional research is required to establish a potential link between progesterone and these immunologic and hemostasis-related effects in both female and male ME/CFS patients.

Sexual dimorphism linked to glucocorticoids significantly influences various physiological processes, encompassing both the immune response [37, 38] and metabolism [39], both of which undergo alterations in ME/CFS [40–43]. Cortisol, the principal glucocorticoid governing these processes under stress, is subject to modulation interactions with sex steroid hormones [39]. Interestingly, ME/CFS patients have been reported to exhibit reduced cortisol levels [15, 44], which is associated with documented dysfunction of the hypothalamic-pituitary-adrenal (HPA) axis in a substantial proportion of ME/CFS cases [45]. However, existing evidence suggests that this dysfunction occurs at a central level, likely due to corticotropin-releasing hormone (CRH) deficiency rather than a primary adrenal insufficiency [46]. Consequently, our findings of elevated levels of 11-deoxycortisol in female ME/CFSsa patients and decreased levels of both cortisol and corticosterone in male ME/CFSmm patients offer novel perspectives for further investigating the intricate interplay between glucocorticoids in different ME/CFS subtypes.

In response to stress, the adrenal glands secrete both cortisol and progesterone in women and men [47]. Stress-induced progesterone plays a role in increasing the bioavailability of cortisol by competitively binding to corticosteroid-binding globulin (CBG), thereby reducing the amount of cortisol that can bind to CBG [48]. Additionally, it participates in the negative feedback loop of the HPA axis to regulate the magnitude of the stress response [49]. Progesterone metabolites, particularly allopregnanolone, primarily mediate this inhibitory effect. Allopregnanolone serves as a potent neuroactive steroid that inhibits hypothalamic CRH production [50]. The observed alterations in glucocorticoid and progesterone levels in male ME/CFSmm patients suggest the presence of a negative feedback mechanism. Similar patterns have been proposed in women during the luteal phase of the menstrual cycle, where elevated progesterone and decreased cortisol levels are noted, contrasting with women during the follicular phase who exhibit the opposite pattern [51]. Glucocorticoids play a central role in a broad range of normal and stress-related responses [52]. Reduced glucocorticoid production, relative to progesterone and allopregnanolone levels, may help to elucidate the characteristic dysregulation of physiological functions in ME/CFS, such as diminished physical performance leading to fatigue, pain, and cognitive impairment [45]. Nevertheless, it remains unknown whether this alteration causes the symptoms or if it represents a compensatory mechanism observed in a subset of ME/CFS patients.

The observed elevation in the progesterone/glucocorticoid ratio in ME/CFSmm patients suggests a potential dysregulation in adrenal corticosteroid pathways, considering progesterone's role as a precursor in corticosteroid biosynthesis [5–7]. Our findings point towards the adrenal enzyme P450c11β (11β-hydroxylase; *CYP11B1* gene) as a potential site of dysregulation. This enzyme is responsible for converting 11-deoxycorticosterone to corticosterone and 11-deoxycortisol to cortisol [5–7]. A deficiency in P450c11β activity could contribute to the observed reduction in corticosterone and cortisol levels in male ME/CFSmm patients, as well as the accumulation of 11-deoxycortisol and 17α-hydroxyprogesterone in female ME/CFSsa patients. However, it is important to note that the levels of steroid hormones and other parameters identified in the blood of patients in our cohort do not meet the criteria for classical P450c11β deficiency [52]. Moreover, our findings may be influenced by sex bias due to the presence of extra-adrenal sources of progesterone and 11-deoxycorticosterone in women, specifically the ovaries [53]. While men also produce progesterone from their testicles, it occurs at lower levels [32]. This complexity suggests that the interaction between steroid hormones is more intricate than previously assumed, and consequently, no single hormone may serve as an adequate biomarker. Consistent with these considerations, we achieved a discriminative accuracy of 0.712 and 0.846 for females and males, respectively, in distinguishing between ME/CFS and healthy controls using a two-component PLS-DA that incorporated our set of nine steroid hormones. Further studies should prioritize assessing the potential predictive value of these and other steroid hormones in stratifying ME/CFS patients based on sex and disease severity.

While we observed differences in steroid hormone levels within our ME/CFS patient cohort, stratified by both sex and severity, it is imperative to confirm our findings with a larger sample size. Although our study excluded pregnant women and those within 12 months postpartum or lactating, we recommend incorporating clinical data related to menstrual cycle, menopausal status, pregnancy history, and contraceptive use. This inclusion will enhance our understanding of the impact of ME/CFS on female reproductive health. In agreement with Pollack et al. [54], we certainly consider that this information proves value as it enables the reduction of potential selection bias and data heterogeneity, thereby positively influencing data analysis and interpretability in female ME/CFS patients. Addressing these pertinent aspects will significantly advance the comprehension of ME/CFS pathophysiology and may contribute to the development of personalized therapeutic approaches tailored to specific patient subgroups. Our findings underscore the importance of investigating variations in pathological mechanisms related to severity, with particular attention to severely affected individuals. This subgroup is often underrepresented in ME/CFS research due to limited healthcare access.

### Supplementary Information

Below is the link to the electronic supplementary material.

**Figure S1.** Representative ultra-high performance liquid chromatography-dynamic multiple reaction monitoring (UHPLC-dMRM) chromatograms. **A** Representative UHPLC-dMRM chromatogram of the target steroid hormones and the corresponding internal standards in a plasma sample prepared using supported liquid extraction (SLE). **B** Representative UHPLC-dMRM chromatogram of the targeted steroids and their corresponding internal standards in a calibration-mix with a concentration of 100 ng/ml for the targeted steroid hormones. **Figure S2.** Spearman's correlation coefficient derived from circulating levels of steroid hormones. A matrix encompassing the circulating levels of the nine steroid hormones was employed to calculate the Spearman's correlation coefficient, stratified by sex and disease severity. Participants were categorized into four groups: HC, representing healthy controls; ME/CFSmm, covering mild/moderate cases; and ME/CFSsa, denoting severe ME/CFS patients. Statistical analysis was carried out using R software version 4.0.2. **Figure S3.** Principal component analysis derived from circulating levels of steroid hormones. The scatterplots illustrate the distribution of each study participant in the first/second (**A**) and second/third (**B**) principal components. Participants were classified into three groups: HC, covering healthy controls; ME/CFSmm, representing mild/moderate ME/CFS patients; and ME/CFSsa, indicating severe ME/CFS patients. Statistical analysis was conducted using R software version 4.0.2. **Figure S4.** Predictive analysis for disease status estimation in female and male cohorts. Predictive analysis was conducted to estimate the disease status of each participant in both the female and male cohorts. Different classifiers, including linear discriminant analysis (LDA), random forest (RF), and partial least squares discriminant analysis (PLS-DA), were employed. The set of measured steroid hormones served as respective predictors. Receiver Operating Characteristic (ROC) curves and area under the curve (AUC) were derived to predict the health status of each participant (HC vs. ME/CFS) using the nine steroid hormones as predictors in both the female (A) and male (B) cohorts. (PDF 383 KB)

**Table S1.** Analytical parameters for UHPLC-dMRM of steroid hormones and internal standards. The table presents analytical parameters utilized for UHPLC-dMRM of steroid hormones and internal standards. Details on the employed dMRM transitions, collision energies (CE), fragmentor voltages (F), cell accelerator voltages (CAV), retention times (RT), delta retention times (Δ RT), and polarity modes of the target steroids hormones and corresponding internal standards are provided. **Table S2.** Source parameters for electrospray ionization of targeted steroid hormones and corresponding internal standards. The table provides source parameters employed for electrospray ionization in UHPLC-MS/MS analysis of steroid hormones and internal standards. It includes values for various parameters, encompassing drying gas temperature and flow, nebulizer pressure, sheath gas temperature and flow, and capillary voltage in positive ( +) mode. **Table S3.** Corrected *p* values from multiple testing derived for plasma levels of steroid hormones in female and male ME/CFS patients compared to healthy controls. The table displays *p* values corrected by multiple testing, derived from ANOVA and Kruskal–Wallis tests, for plasma levels of steroid hormones in female and male ME/CFS patients compared to healthy controls. For each test, unadjusted, false discovery rate (FDR)-adjusted, and Benjamini–Yekutieli (BY)-adjusted *p* values were calculated. **Table S4.** *p* values for comparisons of circulating levels of steroid hormones between independent groups in female and male cohorts. The table presents *p* values obtained by comparing two independent groups within both female and male cohorts concerning their circulating levels of steroid hormones. Participants were classified into three groups: HC, denoting healthy controls; ME/CFSmm, representing mild/moderate ME/CFS patients; and ME/CFSsa, indicating severe ME/CFS patients. The displayed *p* values have been adjusted using the Benjamini–Hochberg test. **Table S5.** Estimated *p* values of Jennrich's permutation test for equality of two Spearman's correlation matrices. The table displays *p* values obtained from Jennrich’s permutation test for the equality of two Spearman’s correlation matrices. The presented *p* values encompass matrices 1 and 2, with and without aldosterone data, respectively. This statistical approach was adopted to address missing information in the aldosterone data(PDF 393 KB)

## Data Availability

The dataset used in this study is available upon reasonable request to the corresponding author.

## References

[1] Bateman L, Bested AC, Bonilla HF et al (2021) Myalgic encephalomyelitis/chronic fatigue syndrome: essentials of diagnosis and management. Mayo Clin Proc 96:2861–2878. 10.1016/j.mayocp.2021.07.004 34454716 10.1016/j.mayocp.2021.07.004

[2] Choutka J, Jansari V, Hornig M, Iwasaki A (2022) Unexplained post-acute infection syndromes. Nat Med 28:911–923. 10.1038/s41591-022-01810-6 35585196 10.1038/s41591-022-01810-6

[3] Dafoe W (2021) Extremely severe ME/CFS—a personal account. Healthcare 9:504. 10.3390/healthcare9050504 33925566 10.3390/healthcare9050504 PMC8145314

[4] Fluge Ø, Tronstad KJ, Mella O (2021) Pathomechanisms and possible interventions in myalgic encephalomyelitis/chronic fatigue syndrome (ME/CFS). J Clin Investig. 10.1172/JCI150377 34263741 10.1172/JCI150377 PMC8279575

[5] Miller WL (2017) Steroidogenesis: unanswered questions. Trends Endocrinol Metab 28:771–793. 10.1016/j.tem.2017.09.002 29031608 10.1016/j.tem.2017.09.002

[6] Miller WL, Auchus RJ (2019) The “backdoor pathway” of androgen synthesis in human male sexual development. PLoS Biol 17:e3000198. 10.1371/journal.pbio.3000198 30943210 10.1371/journal.pbio.3000198 PMC6464227

[7] Miller WL, Auchus RJ (2011) The molecular biology, biochemistry, and physiology of human steroidogenesis and its disorders. Endocr Rev 32:81–151. 10.1210/er.2010-0013 21051590 10.1210/er.2010-0013 PMC3365799

[8] Hahner S, Ross RJ, Arlt W et al (2021) Adrenal insufficiency. Nat Rev Dis Primers 7:19. 10.1038/s41572-021-00252-7 33707469 10.1038/s41572-021-00252-7

[9] Chakraborty S, Pramanik J, Mahata B (2021) Revisiting steroidogenesis and its role in immune regulation with the advanced tools and technologies. Genes Immun 22:125–140. 10.1038/s41435-021-00139-3 34127827 10.1038/s41435-021-00139-3 PMC8277576

[10] Klein SL, Flanagan KL (2016) Sex differences in immune responses. Nat Rev Immunol 16:626–638 27546235 10.1038/nri.2016.90

[11] Miwa K (2017) Down-regulation of renin–aldosterone and antidiuretic hormone systems in patients with myalgic encephalomyelitis/chronic fatigue syndrome. J Cardiol 69:684–688. 10.1016/j.jjcc.2016.06.003 27401397 10.1016/j.jjcc.2016.06.003

[12] Boneva RS, Decker MJ, Maloney EM et al (2007) Higher heart rate and reduced heart rate variability persist during sleep in chronic fatigue syndrome: a population-based study. Auton Neurosci 137:94–101. 10.1016/j.autneu.2007.08.002 17851136 10.1016/j.autneu.2007.08.002

[13] Miwa K, Fujita M (2014) Renin–aldosterone paradox in patients with myalgic encephalomyelitis and orthostatic intolerance. Int J Cardiol. 10.1016/j.ijcard.2014.01.043 24485613 10.1016/j.ijcard.2014.01.043

[14] Germain A, Barupal DK, Levine SM, Hanson MR (2020) Comprehensive circulatory metabolomics in ME/CFS reveals disrupted metabolism of acyl lipids and steroids. Metabolites 10:34. 10.3390/metabo10010034 31947545 10.3390/metabo10010034 PMC7023305

[15] Chang C-J, Hung L-Y, Kogelnik AM et al (2021) A comprehensive examination of severely ill ME/CFS patients. Healthcare 9:1290. 10.3390/healthcare9101290 34682970 10.3390/healthcare9101290 PMC8535418

[16] Germain A, Ruppert D, Levine S, Hanson M (2018) Prospective biomarkers from plasma metabolomics of myalgic encephalomyelitis/chronic fatigue syndrome implicate redox imbalance in disease symptomatology. Metabolites 8:90. 10.3390/metabo8040090 30563204 10.3390/metabo8040090 PMC6315598

[17] Blauensteiner J, Bertinat R, León LE et al (2021) Altered endothelial dysfunction-related miRs in plasma from ME/CFS patients. Sci Rep 11:10604. 10.1038/s41598-021-89834-9 34011981 10.1038/s41598-021-89834-9 PMC8134566

[18] Bertinat R, Villalobos-Labra R, Hofmann L et al (2022) Decreased NO production in endothelial cells exposed to plasma from ME/CFS patients. Vascul Pharmacol 143:106953. 10.1016/j.vph.2022.106953 35074481 10.1016/j.vph.2022.106953

[19] Carruthers BM, Jain AK, De Meirleir KL et al (2003) Myalgic encephalomyelitis/chronic fatigue syndrome. J Chronic Fatigue Syndr 11:7–115. 10.1300/J092v11n01_02 10.1300/J092v11n01_02

[20] Fukuda K, Straus SE, Hickie I et al (1994) The chronic fatigue syndrome: a comprehensive approach to its definition and study. Ann Intern Med 121:953–959. 10.7326/0003-4819-121-12-199412150-00009 7978722 10.7326/0003-4819-121-12-199412150-00009

[21] Lacerda EM, Mudie K, Kingdon CC et al (2018) The UK ME/CFS Biobank: a disease-specific biobank for advancing clinical research into myalgic encephalomyelitis/chronic fatigue syndrome. Front Neurol 9:1026. 10.3389/fneur.2018.01026 30564186 10.3389/fneur.2018.01026 PMC6288193

[22] Lacerda EM, Bowman EW, Cliff JM et al (2017) The UK ME/CFS Biobank for biomedical research on myalgic encephalomyelitis/chronic fatigue syndrome (ME/CFS) and multiple sclerosis. Open J Bioresour. 10.5334/ojb.28 28649428 10.5334/ojb.28 PMC5482226

[23] Venables WN, Ripley BD (2002) Modern applied statistics with S. Springer, New York

[24] Liaw A, Wiener M (2002) Classification and regression by randomForest. R News 2:18–22

[25] Kuhn M (2008) Building predictive models in *R* using the caret package. J Stat Softw. 10.18637/jss.v028.i05 10.18637/jss.v028.i05

[26] Robin X, Turck N, Hainard A et al (2011) pROC: an open-source package for R and S+ to analyze and compare ROC curves. BMC Bioinform 12:77. 10.1186/1471-2105-12-77 10.1186/1471-2105-12-77 PMC3068975 21414208

[27] López-Ratón M, Rodríguez-Álvarez MX, Suárez CC, Sampedro FG (2014) OptimalCutpoints: an R package for selecting optimal cutpoints in diagnostic tests. J Stat Softw. 10.18637/jss.v061.i08 10.18637/jss.v061.i08

[28] Jennrich RI (1970) An asymptotic χ^2^ test for the equality of two correlation matrices. J Am Stat Assoc 65:904–912. 10.1080/01621459.1970.10481133 10.1080/01621459.1970.10481133

[29] Xia J, Sinelnikov IV, Han B, Wishart DS (2015) MetaboAnalyst 3.0—making metabolomics more meaningful. Nucleic Acids Res 43:W251–W257. 10.1093/nar/gkv380 25897128 10.1093/nar/gkv380 PMC4489235

[30] Kolde R, Kolde M (2015) Package “pheatmap.” R Package 1:790

[31] Hammes SR, Levin ER (2019) Impact of estrogens in males and androgens in females. J Clin Investig 129:1818–1826. 10.1172/JCI125755 31042159 10.1172/JCI125755 PMC6486327

[32] Oettel M, Mukhopadhyay A (2004) Progesterone: the forgotten hormone in men? Aging Male 7:236–257. 10.1080/13685530400004199 15669543 10.1080/13685530400004199

[33] Kolatorova L, Vitku J, Suchopar J et al (2022) Progesterone: a steroid with wide range of effects in physiology as well as human medicine. Int J Mol Sci 23:7989. 10.3390/ijms23147989 35887338 10.3390/ijms23147989 PMC9322133

[34] Murphy B (2004) Elevated levels of some neuroactive progesterone metabolites, particularly isopregnanolone, in women with chronic fatigue syndrome. Psychoneuroendocrinology 29:245–268. 10.1016/S0306-4530(03)00026-X 14604604 10.1016/S0306-4530(03)00026-X

[35] Swanepoel AC, Visagie A, de Lange Z et al (2016) The clinical relevance of altered fibrinogen packaging in the presence of 17β-estradiol and progesterone. Thromb Res 146:23–34. 10.1016/j.thromres.2016.08.022 27566845 10.1016/j.thromres.2016.08.022

[36] Nunes J, Kruger A, Proal A et al (2022) The occurrence of hyperactivated platelets and fibrinaloid microclots in myalgic encephalomyelitis/chronic fatigue syndrome (ME/CFS). Pharmaceuticals 15:931. 10.3390/ph15080931 36015078 10.3390/ph15080931 PMC9413879

[37] Cain DW, Cidlowski JA (2017) Immune regulation by glucocorticoids. Nat Rev Immunol 17:233–247. 10.1038/nri.2017.1 28192415 10.1038/nri.2017.1 PMC9761406

[38] Taves MD, Ashwell JD (2021) Glucocorticoids in T cell development, differentiation and function. Nat Rev Immunol 21:233–243. 10.1038/s41577-020-00464-0 33149283 10.1038/s41577-020-00464-0

[39] Kroon J, Pereira AM, Meijer OC (2020) Glucocorticoid sexual dimorphism in metabolism: dissecting the role of sex hormones. Trends Endocrinol Metab 31:357–367. 10.1016/j.tem.2020.01.010 32037025 10.1016/j.tem.2020.01.010

[40] Hoel F, Hoel A, Pettersen IKN et al (2021) A map of metabolic phenotypes in patients with myalgic encephalomyelitis/chronic fatigue syndrome. JCI Insight. 10.1172/jci.insight.149217 34423789 10.1172/jci.insight.149217 PMC8409979

[41] Naviaux RK, Naviaux JC, Li K et al (2016) Metabolic features of chronic fatigue syndrome. Proc Natl Acad Sci 113:E5472–E5480. 10.1073/pnas.1607571113 27573827 10.1073/pnas.1607571113 PMC5027464

[42] Mandarano AH, Maya J, Giloteaux L et al (2020) Myalgic encephalomyelitis/chronic fatigue syndrome patients exhibit altered T cell metabolism and cytokine associations. J Clin Investig. 10.1172/jci132185 31830003 10.1172/jci132185 PMC7269566

[43] Uhde M, Indart AC, Green PHR et al (2023) Suppressed immune and metabolic responses to intestinal damage-associated microbial translocation in myalgic encephalomyelitis/chronic fatigue syndrome. Brain Behav Immun Health 30:100627. 10.1016/j.bbih.2023.100627 37396339 10.1016/j.bbih.2023.100627 PMC10308215

[44] de Vega WC, Herrera S, Vernon SD, McGowan PO (2017) Epigenetic modifications and glucocorticoid sensitivity in myalgic encephalomyelitis/chronic fatigue syndrome (ME/CFS). BMC Med Genomics 10:11. 10.1186/s12920-017-0248-3 28231836 10.1186/s12920-017-0248-3 PMC5324230

[45] Papadopoulos AS, Cleare AJ (2012) Hypothalamic–pituitary–adrenal axis dysfunction in chronic fatigue syndrome. Nat Rev Endocrinol 8:22–32. 10.1038/nrendo.2011.153 10.1038/nrendo.2011.153 21946893

[46] Demitrack MA, Dale JK, Straus SE et al (1991) Evidence for impaired activation of the hypothalamic–pituitary–adrenal axis in patients with chronic fatigue syndrome. J Clin Endocrinol Metab 73:1224–1234. 10.1210/jcem-73-6-1224 1659582 10.1210/jcem-73-6-1224

[47] Herrera AY, Nielsen SE, Mather M (2016) Stress-induced increases in progesterone and cortisol in naturally cycling women. Neurobiol Stress 3:96–104. 10.1016/j.ynstr.2016.02.006 27981182 10.1016/j.ynstr.2016.02.006 PMC5146195

[48] Cameron A, Henley D, Carrell R et al (2010) Temperature-responsive release of cortisol from its binding globulin: a protein thermocouple. J Clin Endocrinol Metab 95:4689–4695. 10.1210/jc.2010-0942 20631013 10.1210/jc.2010-0942

[49] Klusmann H, Schulze L, Engel S et al (2022) HPA axis activity across the menstrual cycle—a systematic review and meta-analysis of longitudinal studies. Front Neuroendocrinol 66:100998. 10.1016/j.yfrne.2022.100998 35597328 10.1016/j.yfrne.2022.100998

[50] Crowley SK, Girdler SS (2014) Neurosteroid, GABAergic and hypothalamic pituitary adrenal (HPA) axis regulation: what is the current state of knowledge in humans? Psychopharmacology 231:3619–3634. 10.1007/s00213-014-3572-8 24756763 10.1007/s00213-014-3572-8 PMC4135030

[51] Hamidovic A, Karapetyan K, Serdarevic F et al (2020) Higher circulating cortisol in the follicular vs. luteal phase of the menstrual cycle: a meta-analysis. Front Endocrinol (Lausanne). 10.3389/fendo.2020.00311 32582024 10.3389/fendo.2020.00311 PMC7280552

[52] White PC (2023) Steroid 11β-hydroxylase deficiency and related disorders. In: Genetic steroid disorders. Elsevier, pp 63–79

[53] Nahoul K, Dehennin L, Salat-Baroux J, Scholler R (1988) Deoxycorticosterone secretion by the human ovary. J Steroid Biochem 31:111–117. 10.1016/0022-4731(88)90213-0 3398524 10.1016/0022-4731(88)90213-0

[54] Pollack B, von Saltza E, McCorkell L et al (2023) Female reproductive health impacts of Long COVID and associated illnesses including ME/CFS, POTS, and connective tissue disorders: a literature review. Front Rehabil Sci. 10.3389/fresc.2023.1122673 37234076 10.3389/fresc.2023.1122673 PMC10208411

