# Supplementary figures and images for: Sex and disease severity-based analysis of steroid hormones in ME/CFS
Source: J Endocrinol Invest. 2024 May 10;47(9):2235–48. doi: 10.1007/s40618-024-02334-1 (PMC11369000; doi:10.1007/s40618-024-02334-1)

A

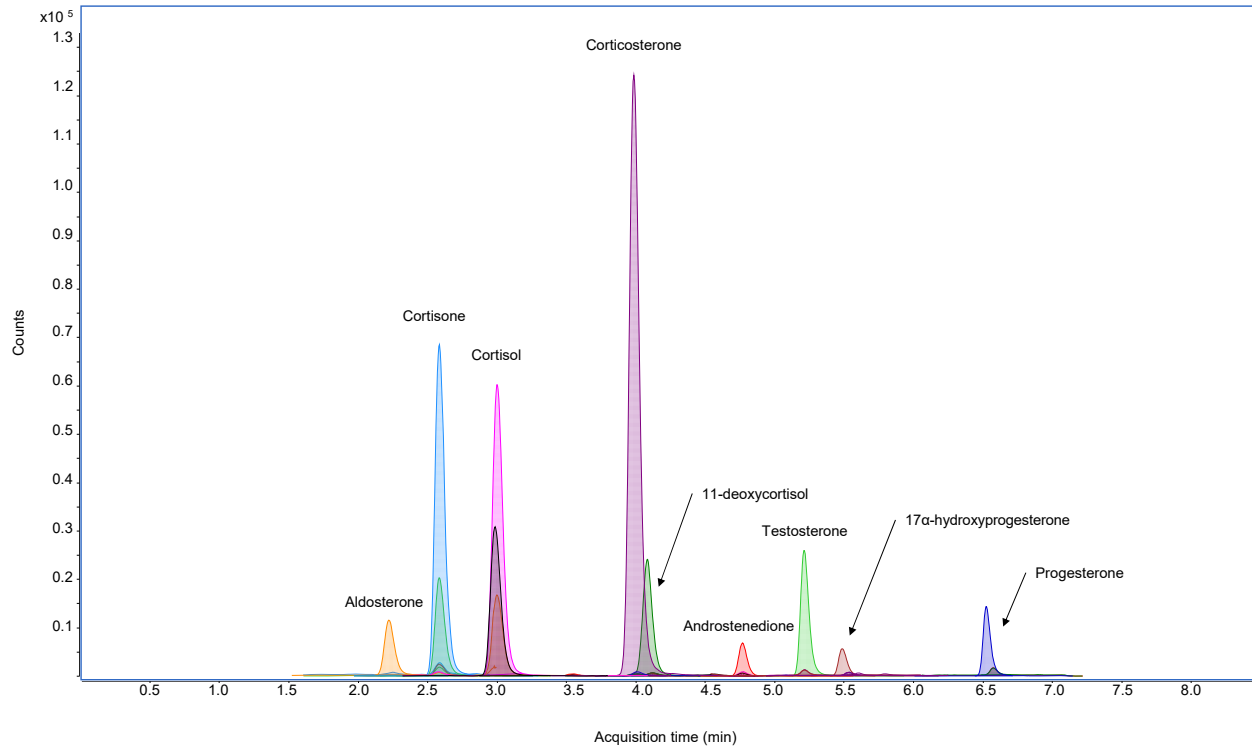

B

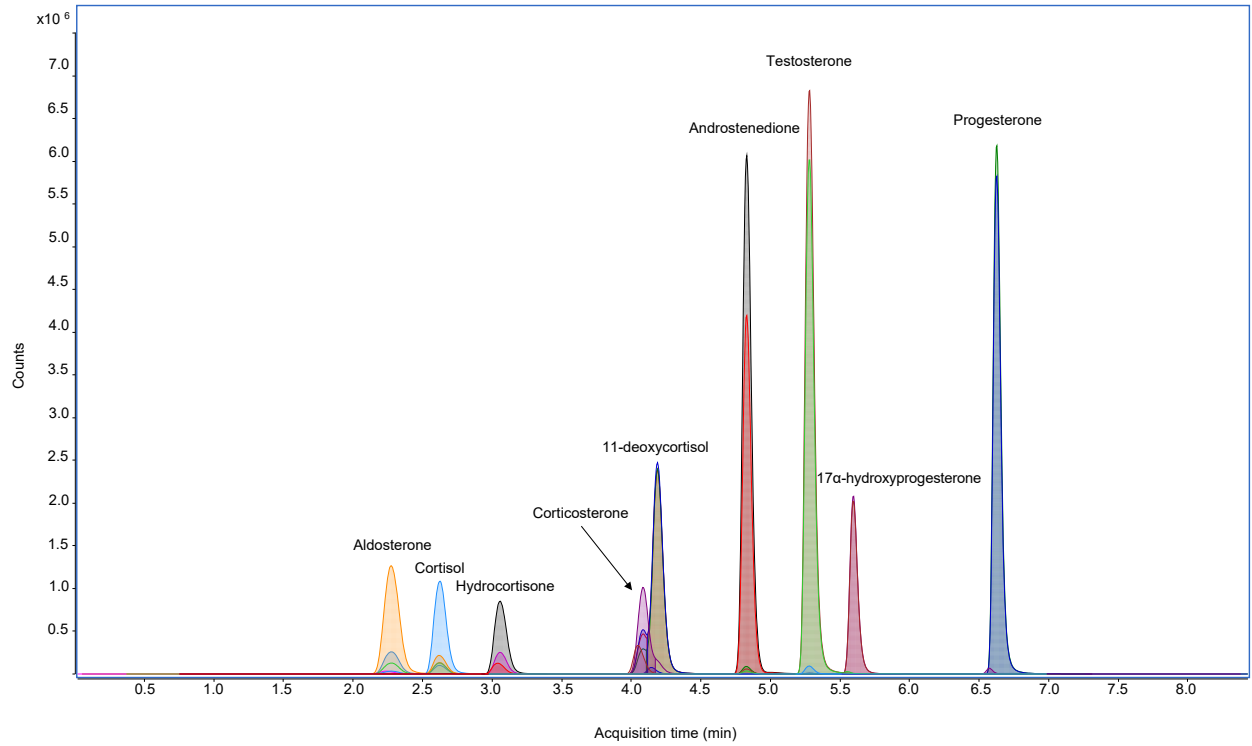

Figure S2

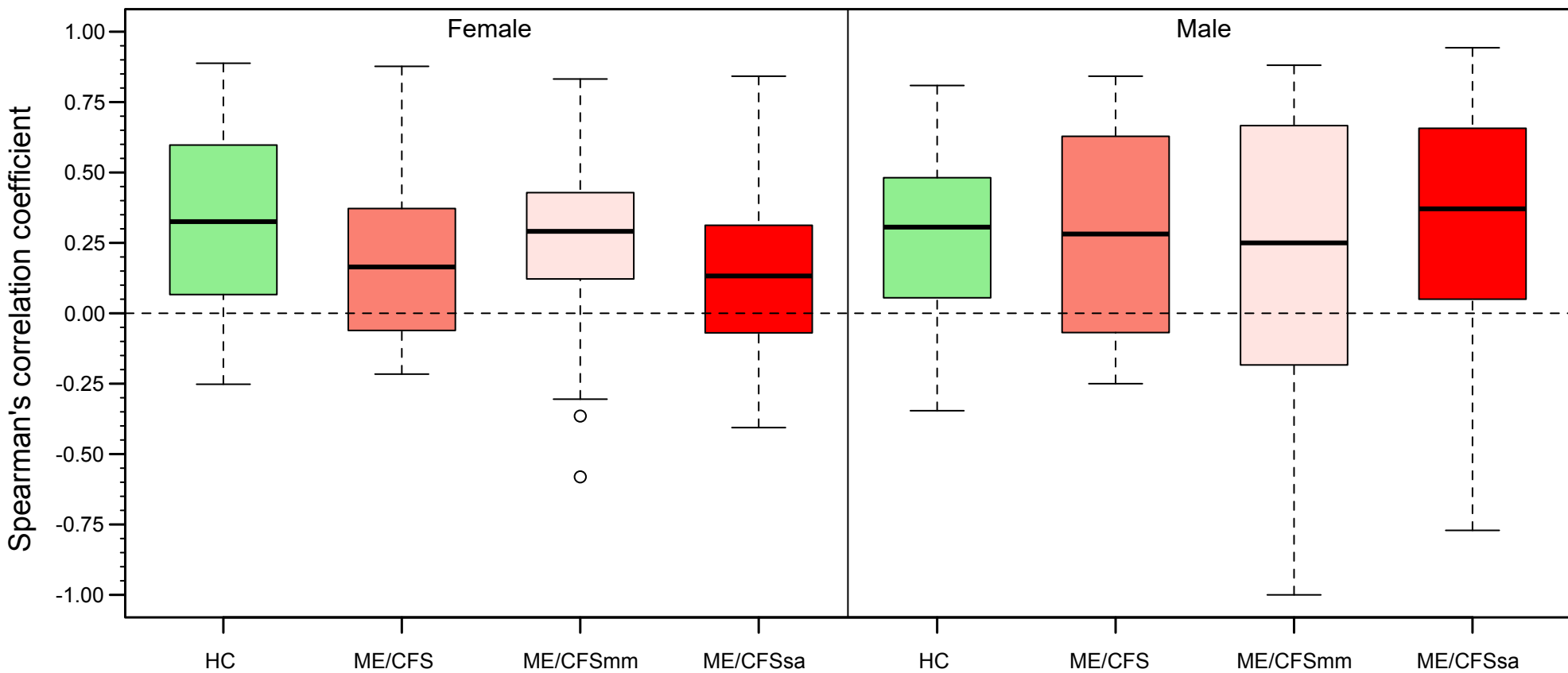

A

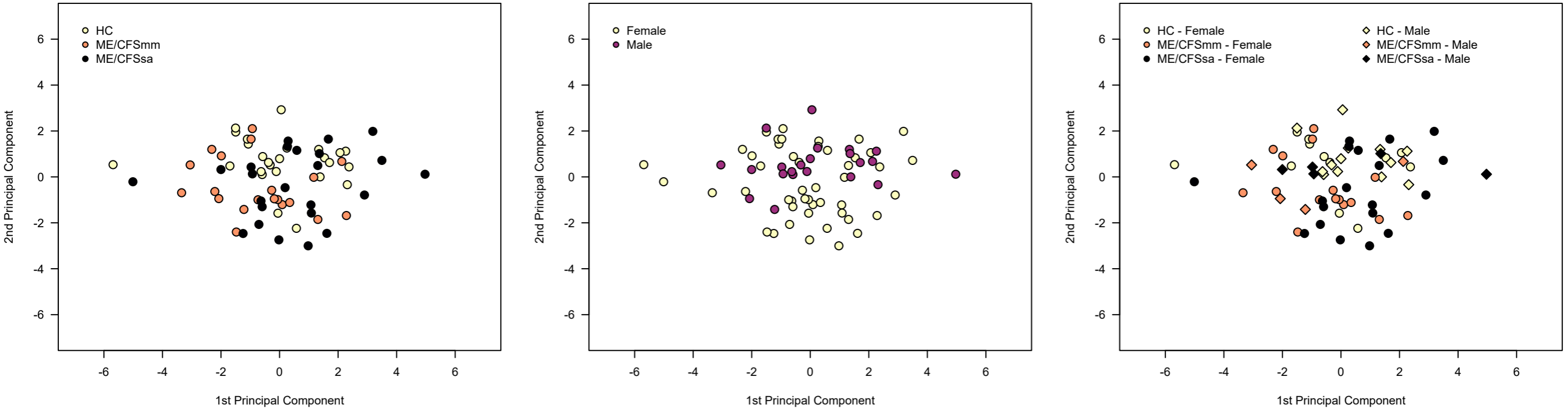

B

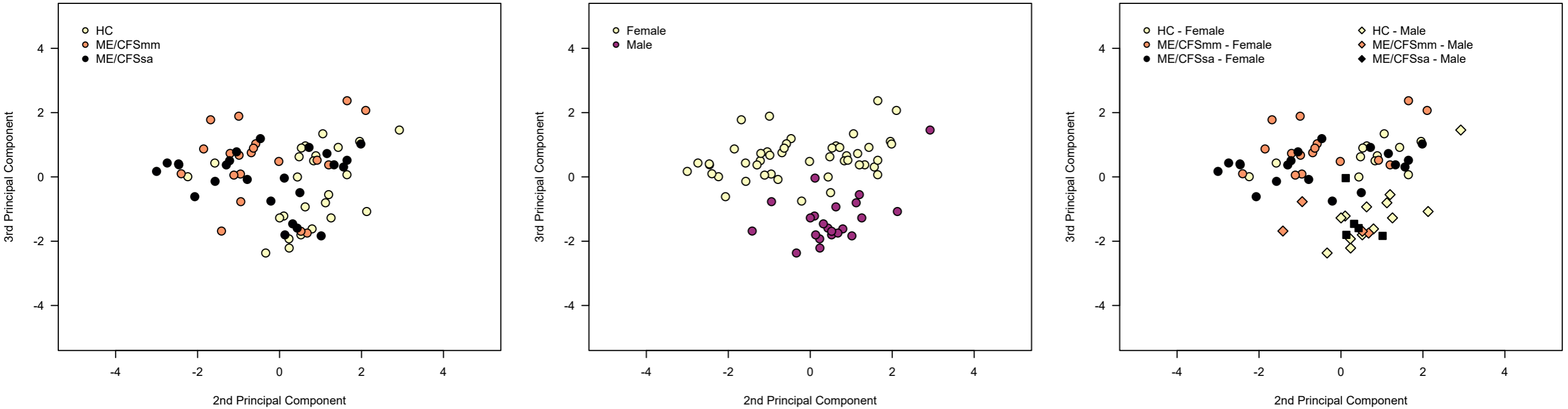

**A****Females**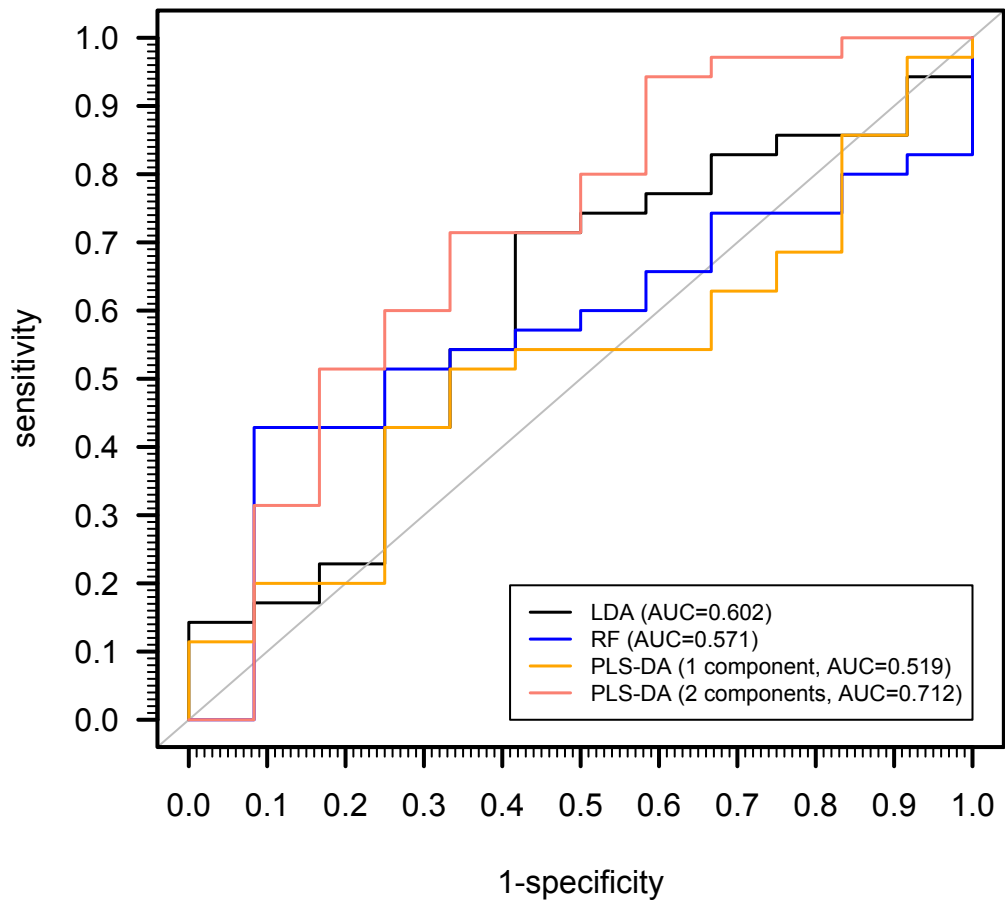**B****Males**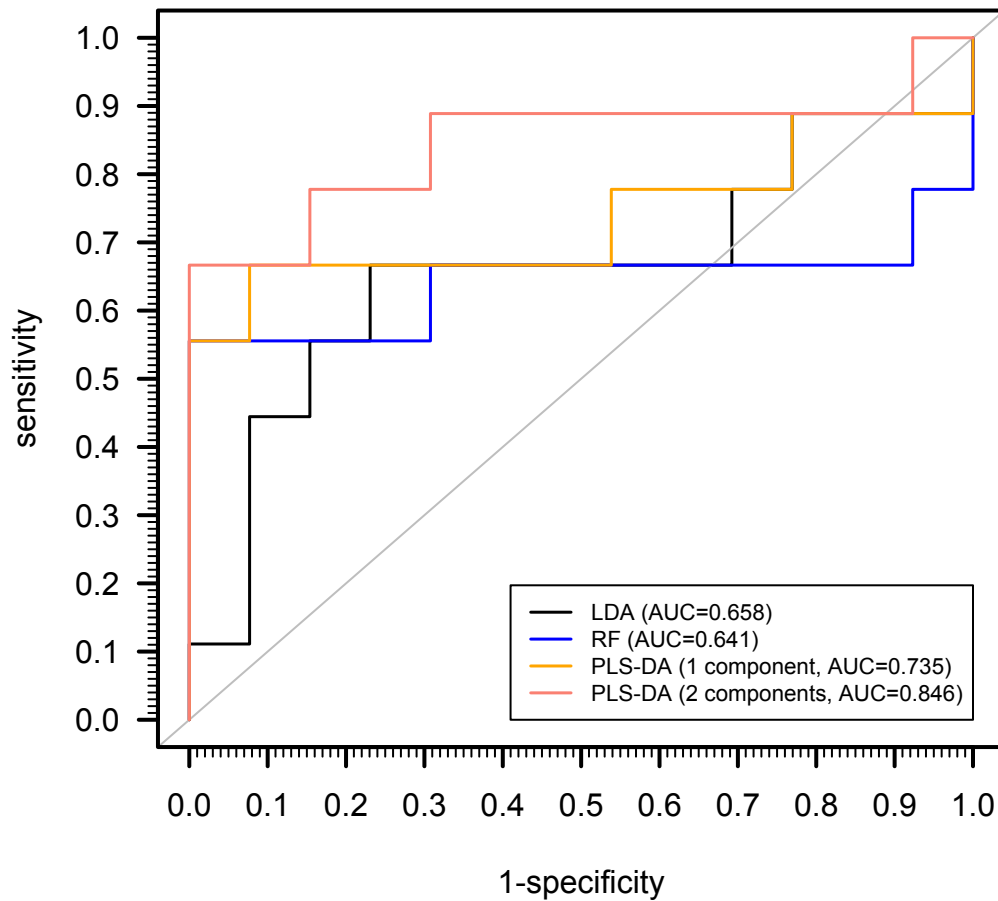

Supplement: Supplementary file 1 — Figure S1. Representative ultra-high performance liquid chromatography-dynamic multiple reaction monitoring (UHPLC-dMRM) chromatograms. A Representative UHPLC-dMRM chromatogram of the target steroid hormones and the corresponding internal standards in a plasma sample prepared using supported liquid extraction (SLE). B Representative UHPLC-dMRM chromatogram of the targeted steroids and their corresponding internal standards in a calibration-mix with a concentration of 100 ng/ml for the targeted steroid hormones. Figure S2. Spearman's correlation coefficient derived from circulating levels of steroid hormones. A matrix encompassing the circulating levels of the nine steroid hormones was employed to calculate the Spearman's correlation coefficient, stratified by sex and disease severity. Participants were categorized into four groups: HC, representing healthy controls; ME/CFSmm, covering mild/moderate cases; and ME/CFSsa, denoting severe ME/CFS patients. Statistical analysis was carried out using R software version 4.0.2. Figure S3. Principal component analysis derived from circulating levels of steroid hormones. The scatterplots illustrate the distribution of each study participant in the first/second (A) and second/third (B) principal components. Participants were classified into three groups: HC, covering healthy controls; ME/CFSmm, representing mild/moderate ME/CFS patients; and ME/CFSsa, indicating severe ME/CFS patients. Statistical analysis was conducted using R software version 4.0.2. Figure S4. Predictive analysis for disease status estimation in female and male cohorts. Predictive analysis was conducted to estimate the disease status of each participant in both the female and male cohorts. Different classifiers, including linear discriminant analysis (LDA), random forest (RF), and partial least squares discriminant analysis (PLS-DA), were employed. The set of measured steroid hormones served as respective predictors. Receiver Operating Characteristic (ROC) cu [file 40618_2024_2334_MOESM1_ESM.pdf]
