# Supplementary material for: Sex and disease severity-based analysis of steroid hormones in ME/CFS
Source: J Endocrinol Invest. 2024 May 10;47(9):2235–48. doi: 10.1007/s40618-024-02334-1 (PMC11369000; doi:10.1007/s40618-024-02334-1)
Supplement: Supplementary file 2 — Table S1. Analytical parameters for UHPLC-dMRM of steroid hormones and internal standards. The table presents analytical parameters utilized for UHPLC-dMRM of steroid hormones and internal standards. Details on the employed dMRM transitions, collision energies (CE), fragmentor voltages (F), cell accelerator voltages (CAV), retention times (RT), delta retention times (Δ RT), and polarity modes of the target steroids hormones and corresponding internal standards are provided. Table S2. Source parameters for electrospray ionization of targeted steroid hormones and corresponding internal standards. The table provides source parameters employed for electrospray ionization in UHPLC-MS/MS analysis of steroid hormones and internal standards. It includes values for various parameters, encompassing drying gas temperature and flow, nebulizer pressure, sheath gas temperature and flow, and capillary voltage in positive ( +) mode. Table S3. Corrected p values from multiple testing derived for plasma levels of steroid hormones in female and male ME/CFS patients compared to healthy controls. The table displays p values corrected by multiple testing, derived from ANOVA and Kruskal–Wallis tests, for plasma levels of steroid hormones in female and male ME/CFS patients compared to healthy controls. For each test, unadjusted, false discovery rate (FDR)-adjusted, and Benjamini–Yekutieli (BY)-adjusted p values were calculated. Table S4. p values for comparisons of circulating levels of steroid hormones between independent groups in female and male cohorts. The table presents p values obtained by comparing two independent groups within both female and male cohorts concerning their circulating levels of steroid hormones. Participants were classified into three groups: HC, denoting healthy controls; ME/CFSmm, representing mild/moderate ME/CFS patients; and ME/CFSsa, indicating severe ME/CFS patients. The displayed p values have been adjusted using the Benjamini–Hochberg test. Table S5. Estim [file 40618_2024_2334_MOESM2_ESM.pdf]

Supplementary Table 1: Analytical parameters for UHPLC-dMRM of steroid hormones and internal standards.

Compound name	Precursor Ion (m/z)	Product ion				F (V)	CAV (V)	RT (min)	Δ RT (min)	Polarity
		Quantifier Ion (m/z)	CE (V)	Qualifier Ion (m/z)	CE (V)					
11-deoxycortisol	347.2	109.0	29	97.0	25	119	3	4.19	1.37	Positive
11-deoxycortisol-[D_5]	352.3	100.1	25	-	25	124	3	4.16	1.32	Positive
17α-hydroxyprogesterone	331.2	109.1	29	97.1	25	114	3	5.60	1.31	Positive
17α-hydroxyprogesterone-[D_8]	339.3	100.1	25	-	25	114	3	5.56	1.25	Positive
Aldosterone	361.2	343.1	13	97.1	37	114	3	2.28	1.35	Positive
Aldosterone-[D_7]	368.2	350.2	13	-	13	124	3	2.26	1.48	Positive
Androstenedione	287.2	97.1	21	109.1	25	119	3	4.83	1.47	Positive
Androstenedione-[$^{13}C_3$]	290.2	100.0	21	-	21	124	3	4.83	1.46	Positive
Corticosterone	347.2	121.0	25	97.1	29	114	3	4.09	0.75	Positive
Corticosterone-[D_4]	351.2	121.0	25	-	25	119	3	4.06	1.35	Positive
Cortisone	361.2	163.1	21	91.0	45	131	3	2.65	1.32	Positive
Cortisone-[$^{13}C_3$]	364.2	166.1	21	-	21	136	3	2.64	1.32	Positive
Cortisol	363.2	105.1	45	121.0	25	119	3	3.08	1.47	Positive
Cortisol-[D_4]	367.2	121.0	21	-	21	124	3	3.06	1.47	Positive
Progesterone	315.2	97.1	21	109.1	25	114	3	6.63	1.16	Positive
Progesterone-[D_9]	324.3	100.1	25	-	25	124	3	6.58	1.15	Positive
Testosterone	289.3	97.1	21	109.1	25	114	3	5.28	1.47	Positive
Testosterone-2,3,4-[$^{13}C_3$]	292.2	100.1	21	-	21	119	3	5.28	1.32	Positive

Supplementary Table 2: Source parameters for electrospray ionization of targeted steroid hormones and corresponding internal standards.

Source parameter	
**Parameter**	**Value (+)**
Gas Temp (°C)	300
Gas Flow (l/min)	10
Nebulizer (psi)	35
Sheath Gas Heater	350
Sheath Gas Flow	10
Capillary (V)	3000
V-Charging	0

Supplementary Table 3: Corrected p-values from multiple testing derived from plasma levels of steroid hormones in female and male ME/CFS patients compared to healthy controls.

Female	ANOVA test			Kruskal-Wallis test		
	Unadjusted p-value	FDR-adjusted p-value	BY-adjusted p-value	Unadjusted p-value	FDR-adjusted p-value	BY-adjusted p-value
Cortisone	0.829	0.829	1.000	0.681	0.766	1.000
Cortisol	0.758	0.829	1.000	0.572	0.736	1.000
Corticosterone	0.324	0.584	1.000	0.312	0.541	1.000
11-deoxycortisol	0.011	0.049	0.138	0.018	0.081	0.228
Aldosterone	0.086	0.194	0.550	0.149	0.336	0.949
Androstenedione	0.585	0.829	1.000	0.361	0.541	1.000
Testosterone	0.736	0.829	1.000	0.792	0.792	1.000
17α-hydroxyprogesterone	0.008	0.049	0.138	0.012	0.081	0.228
Progesterone	0.046	0.139	0.394	0.082	0.246	0.695

Male	ANOVA test			Kruskal-Wallis test		
	Unadjusted p-value	FDR-adjusted p-value	BY-adjusted p-value	Unadjusted p-value	FDR-adjusted p-value	BY-adjusted p-value
Cortisone	0.733	0.824	1.000	0.752	0.846	1.000
Cortisol	0.071	0.213	0.601	0.085	0.256	0.726
Corticosterone	0.015	0.069	0.196	0.035	0.159	0.450
11-deoxycortisol	0.376	0.649	1.000	0.535	0.714	1.000
Aldosterone	0.992	0.992	1.000	0.992	0.992	1.000
Androstenedione	0.433	0.649	1.000	0.295	0.532	1.000
Testosterone	0.649	0.824	1.000	0.556	0.714	1.000
17α-hydroxyprogesterone	0.134	0.302	0.855	0.175	0.394	1.000
Progesterone	0.001	0.005	0.014	0.022	0.159	0.450

Supplementary Table 4: P-values for comparisons of circulating levels of steroid hormones between independent groups in female and male cohorts

**Female**	**HC vs ME/CFSmm**	**HC vs ME/CFSsa**	**ME/CFSmm vs ME/CFSsa**
Cortisone	0.9167	0.9167	0.9614
Cortisol	0.7488	0.7488	0.7488
Corticosterone	0.6868	0.4021	0.4021
11-deoxycortisol	0.9572	0.0276	0.0269
Aldosterone	0.3841	0.3865	0.0970
Androstenedione	0.7260	0.7260	0.7260
Testosterone	0.7568	0.7568	0.7568
17α-hydroxyprogesterone	0.0975	0.0129	0.1025
Progesterone	0.0406	0.1428	0.3229

**Male**	**HC vs ME/CFSmm**	**HC vs ME/CFSsa**	**ME/CFSmm vs ME/CFSsa**
Cortisone	0.7991	0.7991	0.9481
Cortisol	0.0260	0.3584	0.5338
Corticosterone	0.0109	0.8658	0.1371
11-deoxycortisol	0.3941	0.3941	0.3941
Aldosterone	0.9766	0.9766	0.9766
Androstenedione	0.5024	0.9006	0.5024
Testosterone	0.8248	0.6294	0.6294
17α-hydroxyprogesterone	0.1710	0.6014	0.3825
Progesterone	0.0004	0.1202	0.1202

Supplementary Table 5: Estimated p-values of Jennrich's permutation test for equality of two Spearman's correlation matrices.

Cohort	Comparison (vs HC)	P-values	
		Matrix 1	Matrix 2
Female	ME/CFS	0.764	0.718
	ME/CFSmm	0.804	0.289
	ME/CFSsa	0.550	0.799
Male	ME/CFS	0.362	0.631
	ME/CFSmm	0.080	0.356
	ME/CFSsa	0.187	0.267
